# Genome-Wide Identification of *OsZIPs* in Rice and Gene Expression Analysis under Manganese and Selenium Stress

**DOI:** 10.3390/genes15060696

**Published:** 2024-05-27

**Authors:** Xiang Zeng, Shaoxia Yang, Feng Li, Yushuang Yao, Zhengwei Wu, Yingbin Xue, Ying Liu

**Affiliations:** 1 Department of Biotechnology, College of Coastal Agricultural Sciences, Guangdong Ocean University, Zhanjiang 524088, China; 2 Department of Agronomy, College of Coastal Agricultural Sciences, Guangdong Ocean University, Zhanjiang 524088, China

**Keywords:** rice, OsZIPs, expression analysis, manganese toxicity stress, selenium toxic stress

## Abstract

Zinc (Zn)- and iron (Fe)-regulating transport-like proteins (ZIPs) are a class of proteins crucial for metal uptake and transport in plants, particularly for Zn and Fe absorption and distribution. These proteins ensure the balance of trace elements essential for plant growth, development, and metabolic activities. However, the role of the rice (*Oryza sativa*) *OsZIP* gene family in manganese (Mn) and selenium (Se) transport remains underexplored. This research conducted an all-sided analysis of the rice *OsZIPs* and identified 16 *OsZIP* sequences. Phylogenetic analysis categorized the *OsZIPs* predominantly within the three subfamilies. The expression levels of *OsZIPs* in rice root and leaf subjected to Mn and Se toxicity stress were examined through quantitative real-time PCR (qRT–PCR). The findings revealed significant differential expression of many *OsZIPs* under these conditions, indicating a potential regulating effect in the response of rice to Mn and Se toxicity. This work lays a foundation for further functional studies of *OsZIPs*, enhancing our understanding of the response mechanisms of rice to Mn and Se toxicity and their roles in growth, development, and environmental adaptation.

## 1. Introduction

Mn (Manganese) is a vital chemical element found in nearly all creatures and plays various vital functions in plants, including as a cofactor of enzymes [1]. Mn has a crucial role in plant processes such as respiratory action, biosynthesis of proteins, activation hormones, and oxygenic photosynthesis [2]. When the soil contains a higher concentration of Mn than the plant requires, it can result in Mn poisoning and noticeable symptoms of leaf damage, such as Mn spots, leaf wrinkling, and chlorosis. Furthermore, Mn poisoning affects the root system and leads to a reduction in lateral roots and dry weight of root [3]. Excessive Mn at the molecular level hampers the assimilation and transport of other important elements, such as magnesium (Mg), calcium (Ca), phosphorus (P), and iron (Fe) [4]; inhibits chlorophyll synthesis [5]; and reduces the rate of photosynthesis [6].

To maintain a proper balance between the uptake, utilization, and storage of microelements, plant cells have evolved diverse transport networks [7,8]. At the cellular level, ion transporters can regulate gene expression and protein activity in response to varying nutritional conditions [9]. Examples of these transporters include cationic diffusion accelerator protein (CDF), heavy metal ATPase (HMA), and Zn/Fe regulated transporter-like protein (ZIP) [10]. Recent advancements have shed light on the molecular and transport mechanisms of ZIP families in various domains of life, including prokaryotes, eukaryotes, fungi, animals, and archaea. This family of transporters has been identified and characterized for its involvement in metal uptake and transportation [11,12]. Plant ZIP proteins have an important function in the assimilation of iron, zinc, and manganese from soil and are distributed throughout the entire plant [13]. These ZIP proteins maintain the homeostasis of metal ions by facilitating cation transport into the cytosol [14]. The founding family members of the ZIPs include the zinc-regulated transporter (ScZrt1/2) from *Saccharomyces cerevisiae* and the iron-regulated transporter (AtIRT1) from *Arabidopsis thaliana* [13]. Functional studies using yeast have demonstrated that ZIP proteins can transport various bivalent cations, such as Fe^2+^, Zn^2+^, Mn^2+^, Cd^2+^, Co^2+^, Cu^2+^, and Ni^2+,^ from extracellular or intracellular compartments [15,16]. With advancements in whole-genome sequencing and bioinformatics, diverse types of ZIP proteins have been discovered in different plant species. In all, 16 ZIP genes have been authenticated in *A. thaliana* [17], 14 ZIP genes in *Vigna unguiculata* [18], and 58 ZIP genes in wheat (*Triticum aestivum*), 44 of which are homologous to rice ZIP proteins [19]. In potato (*Solanum tuberosum*), 29 ZIP genes have been identified [20]

Selenium (Se) is a nonmetallic element classified in Group 16 of the periodic table along with sulfur (S), due to its similar chemical properties [21]. Se exhibits various biological activities, including immune system support, antioxidant effects, antiviral properties, and anticancer potential [22]. Additionally, Se can enhance plant growth and shield crops against certain biological and environmental stresses, such as drought, salinity, and heavy metal exposure. However, Se can be toxic at relatively high levels, and there is a narrow threshold between Se sufficiency and excess Se compared to other micronutrients, resulting in global concerns regarding Se toxicity [23,24]. Given the similarities between Se and S, their biochemical properties are closely related [25]. Within plant roots, Se is transported across the cell membrane in the form of selenates via sulfate penetrases and channels or as selenites through phosphate transport mechanisms and other ion channels [26]. Alterations in the activity of these transporters across different plant species can affect Se absorption [27]. Previous studies have shown that selenium may affect the accumulation of cations involved in various aspects of cellular oxidative regulation [28]. Se mainly affects enzymatic antioxidant activity [29,30]. Metalloproteins contain one or more metal cations, such as Fe, zinc (Zn), and manganese (Mn) and copper (Cu) ions, which play a key role in catalyzing REDOX reactions [28]. Selenates tend to increase the accumulation of Mg, Zn, and Mn, while selenate/selenite mixtures tend to decrease the accumulation of Ca, Mg, Zn, and Mn, and only iron accumulation is unaffected by selenium [28]. There is an interaction between Se and Mn. For example, selenium can improve the antioxidant performance of Mn toxicity in sunflower (*Helianthus annuus*) seedlings, which may down-regulate the Mn-induced oxidative damage by inhibiting the production of ROS and indirectly down-regulate the antioxidant system [31]. In soybean (*Glycine max*), Se content in root was significantly increased under the treatment of high Mn concentration (100 μM Mn) [32]. Although many reports have elucidated the protective role of selenium under abiotic stress conditions, the function of selenium in abiotic stress response has been unclear. Further research is needed to be sure.

Rice (*O. sativa*) is a primary cereal crop in Asian countries and represents the staple food for 60% of China’s population [33]. Compared to other grain crops, rice has a greater tendency to absorb and accumulate heavy metals [33]. Rice is abundant in antioxidants, carbohydrates, minerals, proteins, and vitamins and has an important part in the human diet and health [34]. Furthermore, rice significantly contributes to global food security and economic development [35]. Ongoing research on rice emphasizes genetic enhancement [36], optimization of tillage techniques [37], strategies for pest resistance [38], and adaptation to environmental changes [39]. Nonetheless, the problem of Mn toxicity poses a significant challenge in rice cultivation, particularly in acidic soils [5]. Mn toxicity disrupts all kinds of physiological responses in vegetable cells, such as stimulating oxidative stress response, suppressing the activity of enzymes, hampering chlorophyll production and oxygenic photosynthesis, and hindering the absorption and transport of other ions, including P, Fe, and Mg [5]. Several studies have reported the Mn toxic effects on different terrestrial plants. For instance, maize (*Zea mays*) exhibits signs of Mn toxicity when Mn accumulation reaches 200 μg [40]. Similarly, rice leaves exhibit light green and brown discoloration when treated with 200 µM Mn, resulting in reduced total dry weight, particularly in the leaf sheath and root system [41]. Molnar et al.’s study demonstrated that both 50 µM and 100 µM Se cause severe root damage in *A. thaliana*, whereas in *Brassica juncea*, only 50 µM Se led to a reduction in fresh weight, but 100 µM sodium selenite resulted in significant decreases in fresh weight and dry weight [42]. Rice variety Xiangyaxiangzhan was treated with 50 µM sodium selenite to inhibit the activity of antioxidant enzymes, and led to excessive production of MDA (malonaldehyde) [43]. Soil acidification is a major problem affecting the global agricultural system [44]. Excessive accumulation of manganese in soil may cause toxicity and is considered to be a major obstacle to plant growth in acidic soils [45]. Selenosis is prevalent in the diet of many countries around the world [46], while in some regions, selenium toxicity is due to natural and man-made events [47]. Therefore, understanding the mechanisms underlying Mn and Se tolerance in rice is crucial for mitigating the adverse effects of Mn and Se toxicity, enhancing rice stress resistance, increasing yield, and ensuring food safety. Nonetheless, the exact role of the rice ZIP gene family in the response to Mn and Se toxicity stress remains unclear.

The function of the *OsZIPs* in the rice genome has yet to be fully clarified. Although it has been reported that OsZIPs have a regulatory effect on metal ions, such as Mn, due to the interaction between Se and Mn and other metal ions, there may be a direct or indirect relationship between Se and OsZIPs, which is still unclear. This study employed multiple analysis methods to identify *OsZIPs* in the rice genome. Analyzing the basic information of *OsZIPs* can aid in elucidating the molecular regulatory mechanism of rice responses to Mn and Se toxicity stress. This study also provides new insights into the survival mechanism of rice under stress conditions such as excessive Mn and Se. Furthermore, a comprehensive analysis of the gene function of *OsZIPs* can provide a theoretical basis for molecular breeding in rice. This is advantageous for enhancing stress resistance and yield, both of which contribute to increasing the economic value of rice. Additionally, this research plays a crucial role in ensuring food security and sustainable agricultural development.

## 2. Materials and Methods

### 2.1. OsZIP Genes Structure Analysis

The genes of *OsZIP1*–*OsZIP10* are quoted from the research report of Huang et al. [48]. *OsZIP11*–*OsZIP16* was found through the Joint Genome Institute (JGI) project Phytozome (https://phytozome-next.jgi.doe.gov/) (accessed on 1 April 2024), CRDC (China Rice Data Center) (https://www.ricedata.cn/gene/) (accessed on 1 April 2024) and the NCBI (National Center for Biotechnology Information) (https://www.ncbi.nlm.nih.gov/) (accessed on 1 April 2024) Sequence alignment and screening. InterPro database (https://www.ebi.ac.uk/interpro/) (accessed on 1 April 2024) was used to complete the structural domain comparison of OsZIP1–OsZIP16 and further confirmation. Chromosomal localization was analyzed using Tbtools software (v1.098) [49], and CDS (codon sequence) size and protein length information were provided by Phytozome. MW (kda) and PI were analyzed by Sequence Manipulation Suite website (https://www.detaibio.com/sms2/index.html) (accessed on 1 April 2024). Subcellular localization was implemented by using WoLF PSORT website (https://www.genscript.com/wolf-psort.html) (accessed on 1 April 2024).

Genetic structure analysis of the *OsZIPs* was performed using the Gene Structure Display Server (GSDS) website (http://gsds.gao-lab.org/) (accessed on 2 April 2024) on account of annotation information from the Rice Genome gff (https://phytozome-next.jgi.doe.gov/info/Osativa_v7_0) (accessed on 3 April 2024). A structure model of rice ZIPs was established. Furthermore, Toolkit for Biologists, integrating various biological data processing tools (TBtools), was utilized to make the structure concrete and visually represent the prediction outcomes [49].

### 2.2. Construction of the ZIP Phylogenetic Tree

The sequences of the amino acid in *OsZIPs* were compared with those of soybean (*G. max*), *A. thaliana*, and maize (*Z. mays*) (Table S1 for the sequence used) using MEGA11 (Molecular Evolutionary Genetics Analysis version 11) software [50]. A phylogenetic tree of the *OsZIPs* was constructed using the NJ (neighbor-joining) method. The analysis of bootstrap was employed to construct 1000 duplicate NJ trees for phylogenetic analysis of rice ZIPs and other species. The resulting phylogenetic tree was constructed using Evolview Online Tools (EOT) (https://www.evolgenius.info/evolview/#/treeview) (accessed on 2 April 2024).

### 2.3. Chromosome Localization of OsZIPs

Based on the rice genome annotation information obtained from the website of Phytozome (https://phytozome-next.jgi.doe.gov/) (accessed on 2 April 2024), the positions of 16 rice *OsZIP* genes on chromosomes were determined. TBtools was applied to perform chromosome localization [49].

### 2.4. Analysis of OsZIPs Conserved Motif

The sequences of the protein in rice ZIPs were sent to the website of MEME (Multiple Em for Motif Elicitation) (http://meme-suite.org/, accessed on 2 April 2024) [51] for motif prediction. The NCBI CDD Batch domain (https://www.ncbi.nlm.nih.gov/Structure/bwrpsb/bwrpsb.cgi, accessed on 3 April 2024) was used to forecast the structure [52]. The motifs and domains of the rice ZIPs were visualized using TBtools [49]. Additionally, the domain structures of the *OsZIPs* were authenticated via the website of InterPro (https://www.ebi.ac.uk/interpro, accessed on 3 April 2024).

### 2.5. Cis Acting Factor Analysis

The 2 kb upriver promoter region sequences of the *OsZIPs*, based on the available rice genome information, were sent to the website of Plant Care (http://bioinformatics.psb.ugent.be/webtools/plantcare/, accessed on 2 April 2024) for cis-acting element prediction [53]. TBTools was used to simplify and visualize the analysis results [49].

### 2.6. Rice Culture Conditions

The rice variety used in this study is HH-11 (Haihong-11), which was provided by Professor Hongkai Zhou of Guangdong Ocean University in Zhanjiang, China. Rice seeds were germinated in quartz sand for 10 days and then subjected to Mn and Se toxicity treatments. Rice plants with uniform growth were transplanted into 5 L plastic buckets filled with 4 L nutrient solution for hydroponic growth [54]. The NSP1040 nutrient solution (Mn-deficient Yoshida rice nutrient solution) (Coolaber, Beijing, China) was used, and the formula is shown in Table S2 [38]. In the nutrient solution, 10 µM MnSO_4_ was added as the normal growth concentration of rice, whereas 500 µM MnSO_4_ was added as the toxic concentration of Mn [55]. For the rice Se treatment, sodium selenite was added exogenously, whereas for the Se toxicity treatment, the nutrient solution contained 64 µM Se, and for the control, the nutrient solution without Se was used [42]. Each experiment was replicated 3 times. The temperature for regulating rice growth was maintained at 25–27 °C during the day and at 20–22 °C at night. The light cycle was about 12 h per day, with an illumination intensity of 2000 lux, and the hydroponic nutrient solution was replaced every 5 days. The pH of the hydroponic solution was regulated to 5.0 using 1 M NaOH or HCl every 2 days.

### 2.7. Analysis of OsZIP Genes Expression Levels in Rice

After 20 days of Se or Mn toxicity treatment, the rice root and leaf were collected and ground into a fine powder using liquid nitrogen. A MolPure^®^ Plant RNA Kit (19291ES50, 50 T, YEASEN, Shanghai, China) was used to remove proteins, genomic DNA, and pigments from the sample. The RNA was then enriched using an RNA adsorption column, eluted, and collected. The quality and concentration of the RNA were tested via a spectrophotometer of Micro Drop Ultra-Micro (BIO-DL, Shanghai, China). RNA was reverse transcribed into cDNA using Hifair^®^ II1st Strand cDNA Synthesis Kit (11119ES60, 100 T, YEASEN, Shanghai, China). The configured reverse transcription system was 20 μL, including 3 μL 5 × gDNA digester Mix, 2 μL Total RNA, 15 μL RNase-free H_2_O; the reaction condition was 42 °C for 2 min, and then added 4 × Hifair^®^III SuperMix plus in the reaction mixture at 25 °C for 5 min; 55 °C, 15 min; 85 °C for 5 min [56]. Fluorescent quantitative PCR (qRT–PCR) was accomplished by using a fluorescence ration PCR instrument with CFX Connect Optics Module (Bio-Rad, Hercules, CA, USA) [56] to measure the expression quantities of the *OsZIPs*. The reagent of qRT–PCR used was Hieff UNICON^®^ Universal Blue qPCR SYBR Green Master Mix (11184ES08, Yeasen, Shanghai, China). The primers for qRT–PCR were obtained from Guangzhou IGE Biotechnology Co., Ltd. (Guangzhou, China). The cDNA sample was diluted 3 times, and qRT–PCR was performed using a 20 μL reaction system: H_2_O: 8 μL; Mix: 10 μL; Primer F: 0.5 μL; Primer R: 0.5 μL; cDNA: 1 μL. The reaction programs were set up as follows: 95 °C for 3 min, 95 °C for 15 s, 60 °C for 20 s, and 60 °C for 20 s for 45 cycles. The relative transcript level was converted by using *OsActin* (*LOC_Os03g50885*) as the internal parameter by the 2^−ΔΔCT^ method [57]. The qRT–PCR primers are displayed in Table S3. Microsoft Excel 365 (Microsoft Office 2023) (Microsoft Corporation, Redmond, Washington, DC, USA) was used for data analysis and charting, and the data were expressed as the mean and standard deviation (SD) of the three experimental replicates. The *t*-test was used to evaluate the differences between the control group and the toxic-treatment group [56]. 

## 3. Results

### 3.1. Analysis of OsZIPs Basic Information

Sixteen gene paralogs of OsZIPs were derived from the entire rice genome. Five of these genes were situated on chromosome 05, three genes on chromosomes 03 and 08, and the remaining five genes on chromosomes 01, 02, 04, 06, and 07 (Table 1). The average length of CDS in *OsZIPs* was about 1127 bp, with the longest gene, *OsZIP14*, being 1497 bp and encoding a protein of 498 amino acids (Table 1). The molecular weights of the *OsZIPs* fell between 24.15 and 53.58 kDa, and the isoelectric points fell between 6.07 and 8.89. Subcellular localization predictions revealed that *OsZIP10* was located in chloroplasts, whereas the remaining 15 *OsZIPs* were located in cell membranes (Table 1).

### 3.2. OsZIPs Structure Analysis

**Figure 1:**
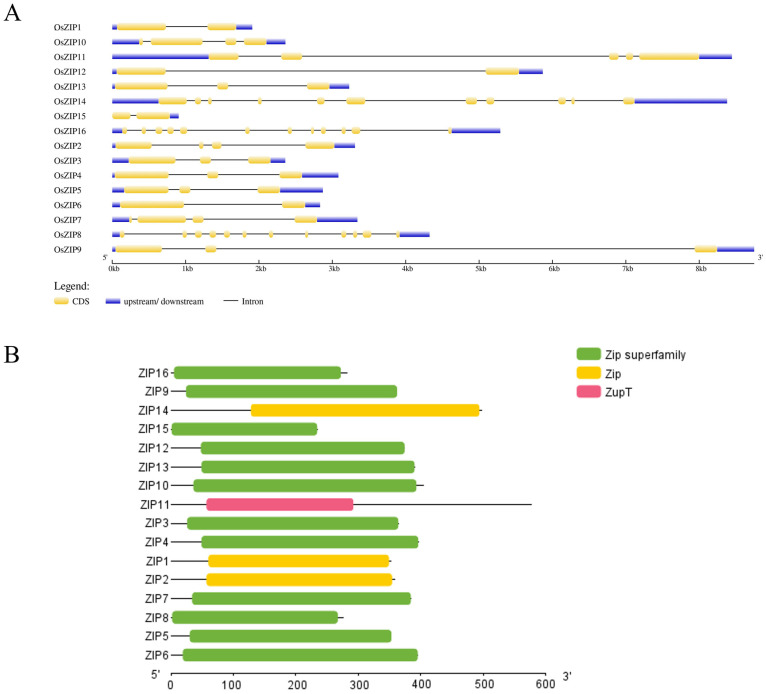
Results of structure and domain analysis of *OsZIPs*: (**A**) Structure of genetics; (**B**) analysis of domain. The conserved protein domain families Zip, the Zip superfamily, and Zup T were classified as models that might span multiple domains.

**Figure 2:**
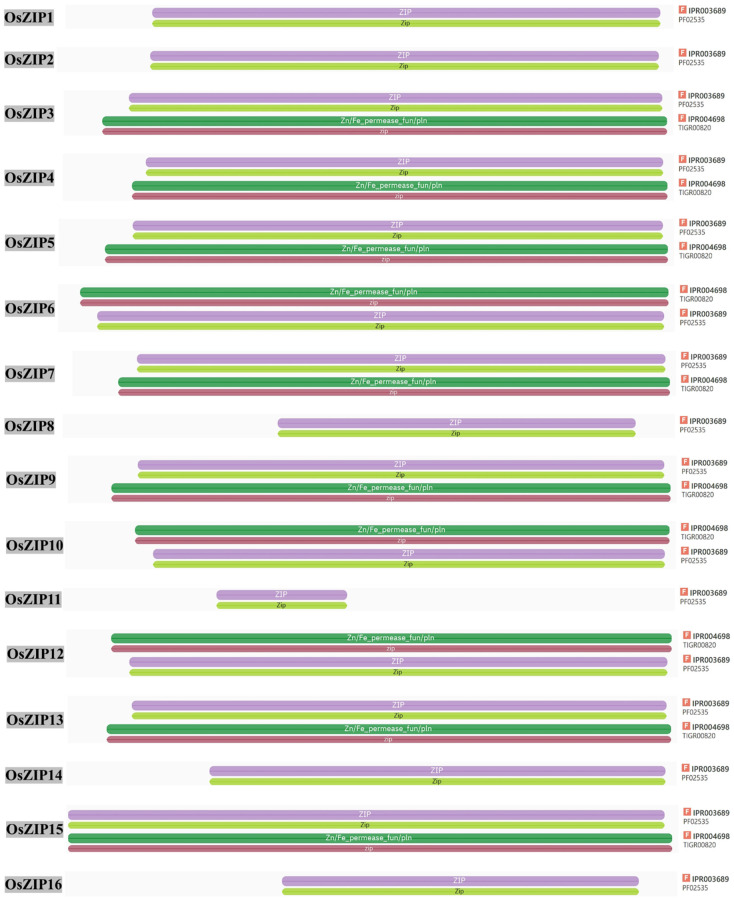
The *OsZIPs* domain was searched by InterPro. All the *OsZIPs* contained the ZIP/Zip domain (number PF02535). F indicates the InterPro domain number.

Sixteen OsZIPs were identified in the rice genome (Table 1). Analysis of their genetic structure revealed that most of the genes were between 2 and 4 kb in length, and most of the genes contained more than three exons (Figure 1A). Domain analysis predicted that 16 genes contained the ZIP/Zip and Zup T domains (Figure 1B). InterPro was used to analyze the domain of OsZIPs protein sequences, and it was found that 16 OsZIPs contained ZIP/zip domains, which were numbered as IPR003689 (PF02535); OsZIP3, OsZIP4, OsZIP5, OsZIP6, OsZIP7, OsZIP9. OsZIP10, OsZIP12, OsZIP13, and OsZIP15 also contained Zn/Fe-permease-fun/pin/zip domains, numbered IPR004698 (TIGR00820) (Figure 2).

### 3.3. Phylogenetic Tree of ZIPs

**Figure 3:**
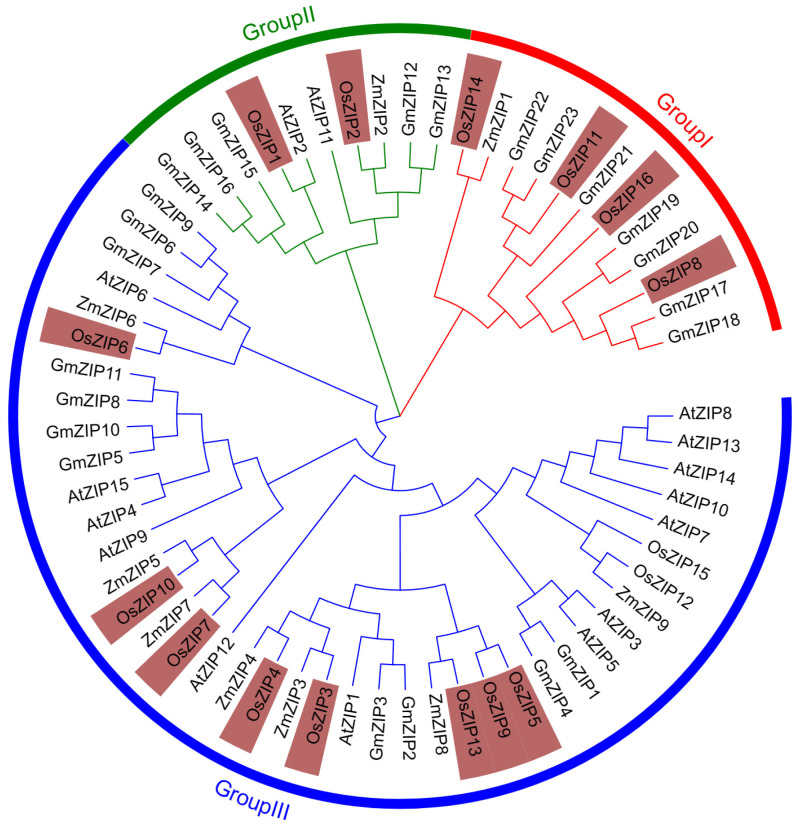
A phylogenetic tree was constructed using ZIPs from *G. max*, *O. sativa*, *Z. mays*, and *A. thaliana*. *O. sativa*: OsZIP1 (LOC_Os01g74110.1), OsZIP2 (LOC_Os03g29850.1), OsZIP3 (loc_OS04G52310.11), OsZIP4 (LOC_Os08g10630.1), OsZIP5 (LOC_Os05g39560.1), OsZIP6 (loc_OS05G072101.1), OsZIP7 (loc_OS05G10940.01), OsZIP8 (LOC_Os02g10230.1), OsZIP9 (LOC_Os05g39540.1), OsZIP10 (LOC_Os06g37010.1), OsZIP11 (loc_OS05G25194-1), OsZIP12 (LOC_Os03g46470.1), OsZIP13 (LOC_Os07g12890.1), OsZIP14 (LOC_Os08g36420.1), OsZIP15 (LOC_Os03g46454.1), OsZIP16 (LOC_Os08g01030.1); *A. thaliana*: AtZIP1 (AT3G12750.1), AtZIP2 (AT5G59520.1), AtZIP3 (AT2G32270.1), AtZIP4 (AT1G10970.1), AtZIP5 (AT1G05300.1), AtZIP6 (AT2G30080.1), AtZIP7 (AT2G04032.1), AtZIP8 (AT5G45105.2), AtZIP9 (AT4G33020.1), AtZIP10 (AT1G31260.1), AtZIP11 (AT1G5590.1), AtZIP12 (AT5G62160.1), AtZIP13 (AT4G19690.2), AtZIP14 (AT4G19680.2), AtZIP15 (AT1G60960.1); *G. max*: GmZIP1 (Glyma.20g063100), GmZIP2 (Glyma.08g164400), GmZIP3 (Glyma.15g262800), GmZIP4 (Glyma.13g004400), GmZIP5 (Glyma.17g228600), GmZIP6 (Glyma.11g169300), GmZIP7 (Glyma.14g196200), GmZIP8 (Glyma.04g051100), GmZIP9 (Glyma.18g060300), GmZIP10 (Glyma.14g094900), GmZIP13 (Glyma.18g078600), GmZIP14 (Glyma.15g036200), GmZIP15 (Glyma.15g036300), GmZIP16 (Glyma.13g338300), GmZIP17 (Glyma.13g340900), GmZIP18 (Glyma.15g033500), GmZIP19 (Glyma.11g132500), GmZIP20 (Glyma.12g056900), GmZIP21 (Glyma.16g221000), GmZIP22 (Glyma.09g271900), GmZIP23 (Glyma.18g217100); *Z. mays*: ZmZIP1 (NM_001137726), ZmZIP2 (NM_001159169), ZmZIP3 (NM_001155536), ZmZIP4 (HM048832), ZmZIP5 (NM_001154257), ZmZIP6 (NM_001156151), ZmZIP7 (NM_001157018), ZmZIP8 (NM_001154769), ZmZIP9 (NM_001158638).

With the assistance of MEGA11, the phylogenetic tree of 63 ZIP genes from Glycine max, Oryza sativa, Zea mays, and Arabidopsis thaliana was constructed by the NJ (neighbor-joining) method. According to the phylogenetic tree, plant ZIP genes can be categorized into three subfamilies: subfamily I, subfamily II, and subfamily III. Subfamily I consists of at least 12 proteins, subfamily II consists of at least 10 proteins, and subfamily III consists of a maximum of 41 proteins. Rice ZIP proteins are found in all three subfamilies (Figure 3).

### 3.4. Analysis of the Conserved Motif of Rice ZIPs

**Figure 4:**
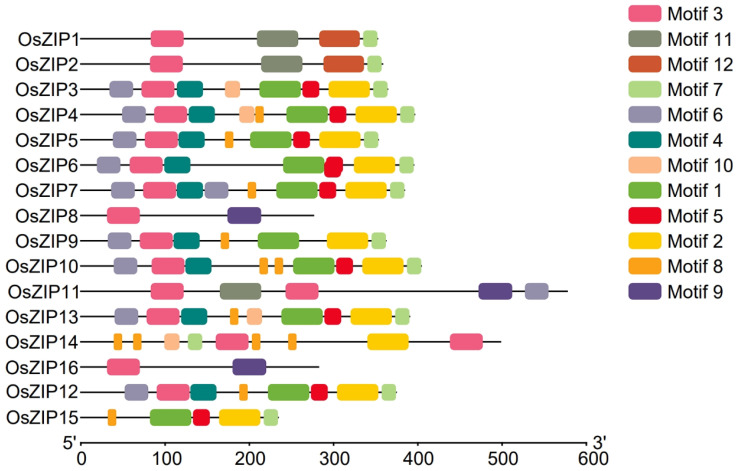
Conserved motifs of rice ZIPs. All genes except *OsZIP15* contained motif 3.

Next, the conserved motifs of the rice ZIP sequences were analyzed via the MEME tool. Most conserved OsZIP motifs had a number of 8–9. All genes, except for *OsZIP15*, contained motif 3, indicating the significance of this motif in the ZIP proteins coding sequence. In addition, there were 9 genes containing motifs 1, 2, 3, 4, 5, 6, 8, and 10 genes containing motif 1, and 11 genes containing motif 2. Thus, these areas might play an important role (Figure 4). However, the specific function and potential role of these motifs need to be further studied.

### 3.5. Chromosome Localization of Rice ZIPs

**Figure 5:**
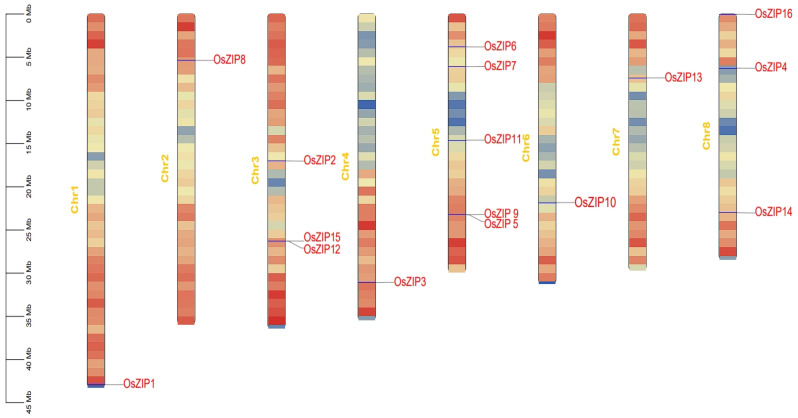
Location of *OsZIPs* on rice chromosomes: *OsZIP1*, *OsZIP2*, *OsZIP3*, *OsZIP5*, *OsZIP6*, *OsZIP7*, *OsZIP8*, *OsZIP9*, *OsZIP10*, *OsZIP11*, *OsZIP12*, *OsZIP13*, *OsZIP14*, *OsZIP15*, and *OsZIP16* were located in gene-dense areas (red and yellow); *OsZIP4* was located in the gene dispersion region (blue).

Further analysis revealed the chromosomal localization of the rice ZIP genes. Mapping revealed that most genes were located in gene-dense regions (indicated in red), whereas *OsZIP4* was located in gene-dispersed regions (indicated in blue). Chromosome 05 harbored four genes, whereas chromosomes 03 and 08 each contained three genes. The remaining chromosomes contained only one ZIP gene each (Figure 5).

### 3.6. Verifying of OsZIP Cis Acting Elements

**Figure 6:**
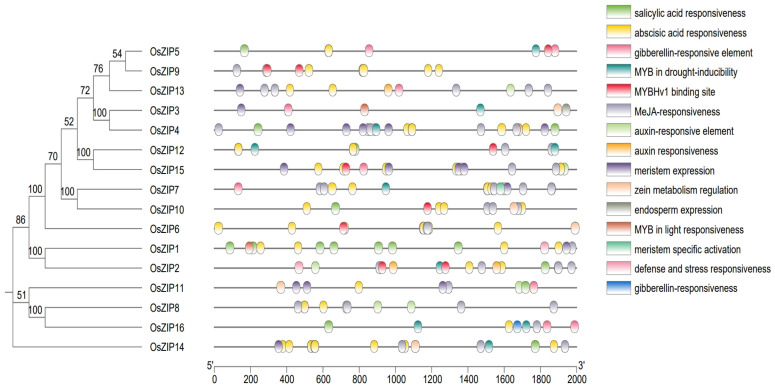
Analysis of cis-acting elements in the *OsZIP* promoters. The 2000 bp region of the OsZIP promoters was used for analysis.

Two-kilobase upstream sequences were chosen from each of the 16 *OsZIP* promoters. Cis-elements within the *OsZIP* promoters were forecasted via the website of Plant CARE. Among all the rice genes, 1–7 abscisic acid response elements were identified (Figure 6). Furthermore, the rice ZIP gene promoter region predominantly comprises methyl jasmonate (MeJA) response elements and salicylic acid response factors. Additionally, MYB binding site response elements were present along with various regulatory factors, including auxin response factors, cryogenic response factors, meristem expression factors, circadian rhythm control factors, cell cycle regulatory factors, and stress and defense response factors (Figure 6).

### 3.7. Analysis of OsZIPs Expression under Mn Poisoning

**Figure 7:**
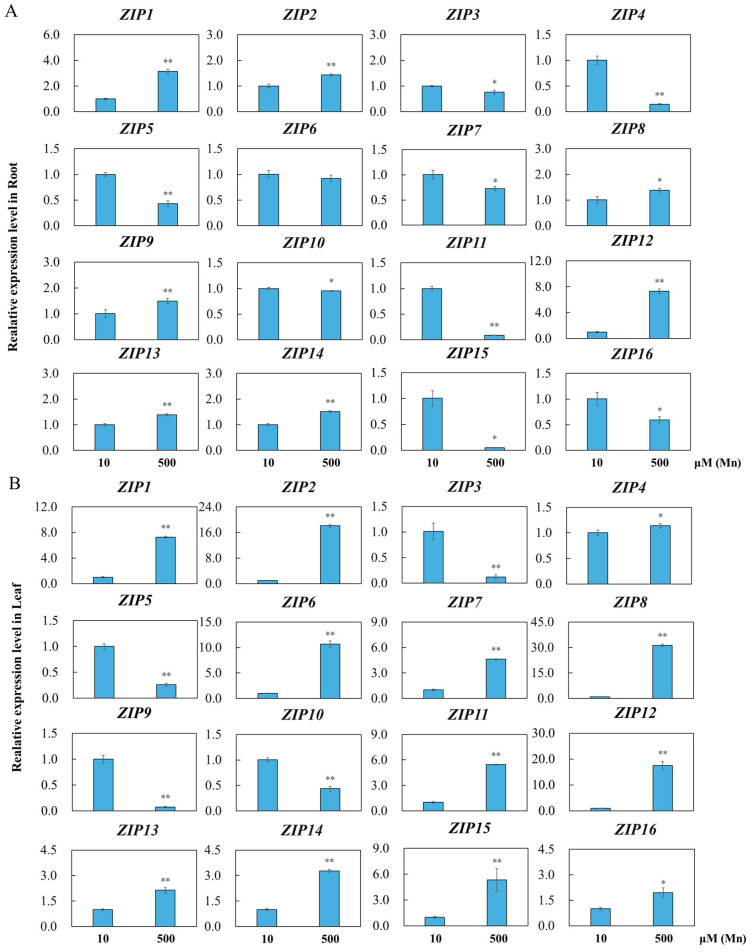
Results of qRT–PCR test of 16 *OsZIPs* in rice leaf and root with 10 and 500 μM Mn treatment. The relative expression levels of *OsZIPs* in the root (**A**) and leaf (**B**) of rice plants were calculated and are presented as the average value and standard deviation (SD) of three experimental replicates. A *t*-test was applied to determine the differences between the control group and Mn toxicity group, with * indicating *p* < 0.05 and ** indicating *p* < 0.01.

Finally, the expression of *OsZIPs* under Mn poisoning was analyzed. *OsZIPs* in rice root and leaf treated with 10 or 500 μM Mn were tested via qRT–PCR, and the expression of these 16 *OsZIPs* in root and leaf treated with Mn was further confirmed, as displayed in Figure 7. The expression levels of *OsZIPs* varied in root and leaf of rice plants under Mn poisoning stress. Specifically, the expression of *OsZIP1*, *OsZIP2*, *OsZIP8*, *OsZIP9*, *OsZIP12*, *OsZIP13*, and *OsZIP14* significantly increased in the roots (Figure 7A). On the other hand, *OsZIP3*, *OsZIP4*, *OsZIP5*, *OsZIP7*, *OsZIP10*, *OsZIP11*, *OsZIP15*, and *OsZIP16* exhibited significantly downregulated expression in the roots (Figure 7A). Moreover, in the leaves, *OsZIP1*, *OsZIP2*, *OsZIP4*, *OsZIP6*, *OsZIP7*, *OsZIP8*, *OsZIP11*, *OsZIP12*, *OsZIP13*, *OsZIP14*, *OsZIP15*, and *OsZIP16* were significantly upregulated, whereas *OsZIP3*, *OsZIP5*, *OsZIP9*, and *OsZIP10* were significantly downregulated (Figure 7B).

### 3.8. Analysis of OsZIPs Expression under Se Toxicity

**Figure 8:**
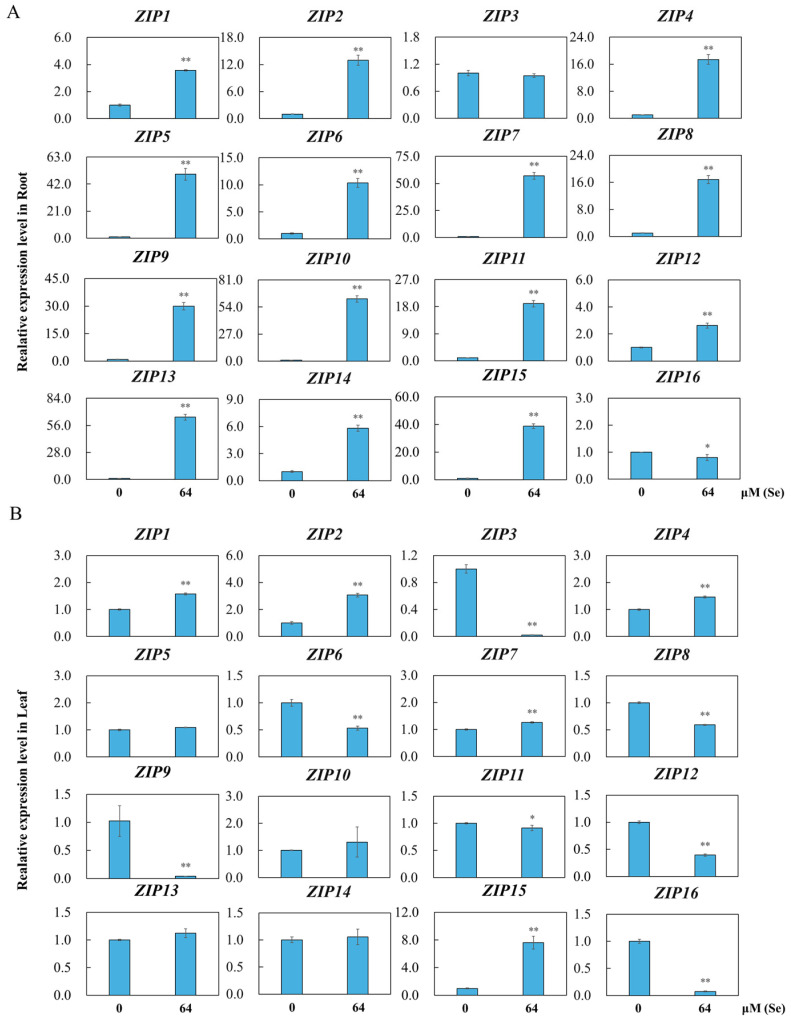
Results of qRT–PCR test of 16 *OsZIPs* in rice leaf and root with 0 and 64 μM Se treatment. The relative expression of *OsZIPs* in rice roots (**A**) and leaves (**B**) was calculated. The relative expression levels of *OsZIPs* in the root (**A**) and leaf (**B**) of rice plants were calculated and are presented as the average value and standard deviation (SD) of three experimental replicates. A *t*-test was applied to determine the differences between the control group and Mn toxicity group, with * indicating *p* < 0.05 and ** indicating *p* < 0.01.

To analyze *OsZIP* expression under Se toxicity in the roots and leaves of rice plants treated with 64 μM Se, qRT–PCR analysis was conducted, and the gene expression results of these 16 *OsZIPs* were further verified, as shown in Figure 8. Under Se toxicity stress, the expression of most *OsZIPs* in the roots was significantly greater than that under Mn toxicity stress. Specifically, *OsZIP1*, *OsZIP2, OsZIP4*, *OsZIP5*, *OsZIP6*, *OsZIP7*, *OsZIP8*, *OsZIP9*, *OsZIP10*, *OsZIP11*, *OsZIP12*, *OsZIP13*, *OsZIP14*, and *OsZIP15* exhibited significantly upregulated expression, whereas only *OsZIP16* exhibited significantly downregulated expression in the roots (Figure 8A). Additionally, in the leaves, *OsZIP1*, *OsZIP2*, *OsZIP4*, *OsZIP7*, and *OsZIP15* were significantly upregulated, whereas *OsZIP3*, *OsZIP6*, *OsZIP8*, *OsZIP9*, *OsZIP11*, *OsZIP12*, and *OsZIP16* were significantly downregulated (Figure 8B).

## 4. Discussion

With advancements in bioinformatics, an increasing number of ZIP members have gradually been discovered in various plant species, including *A. thaliana* [17], cowpea (*Vigna sinensis*) [18], wheat (*T. aestivum*) [19], and potato (*S. tuberosum*) [20]. ZIP transporters are located primarily in the plasma membrane and on the membranes of various intracellular organelles [58]. Research has revealed that the number and distribution of ZIPs vary across plant species. In our study, we identified 16 ZIPs in rice, all of which were located in the cell membrane and chloroplast. Cell membrane permeability is a fundamental characteristic that governs the movement of solutes and solvents into and out of cells or intracellular compartments [59]. This suggests that *OsZIPs* may be involved in the transmembrane transport of ions.

Conserved motif analysis revealed slight variations in the number of conserved motifs in the *OsZIP* protein (Figure 4), suggesting potential functional differences in *the OsZIP* protein. The interpretation of the cis-acting factors of the *OsZIPs* revealed a range of response factors in plant development and stress (Figure 6). Several cis-elements, such as salicylic acid (SA) response factor, auxin response factor, MYB binding site response factor, and gibberellin response factor, were identified among the *OsZIPs*. Furthermore, it included factors related to cell cycle regulation, circadian rhythm control, and low-temperature response. These findings suggest that *OsZIPs* likely have a significant regulative role in plant development, synthesis of hormones, and both abiotic and biotic stress responses in rice.

The analysis of gene expression patterns is crucial for confirming gene function [60]. In the current study, diverse expression levels of *OsZIPs* in the roots and leaves under Mn and Se toxicity stress conditions were observed (Figure 7 and Figure 8). Nearly all the *OsZIPs* showed differential expression in the rice leaves and roots, with a greater number of *OsZIPs* being differentially expressed in root in response to Mn and Se poisoning than in leaf. The expression levels of OsZIP6, OsZIP8, and OsZIP12 in rice leaves were increased by at least 10 times under the treatment of 500 μM Mn, while the expression levels of OsZIPs in rice roots were highly differential under the treatment of 64 μM Se. The expressions of OsZIP11 and OsZIP5, OsZIP7, OsZIP9, OsZIP10, OsZIP13, and OsZIP15 were increased at least 10-fold. These findings suggest that OsZIPs might have different response sites to manganese and selenium toxicity stress. The response of rice to manganese toxicity was mainly in leaf, while the response to selenium toxicity was mainly in root. These findings indicate that *OsZIPs* likely have a vital regulatory function in the response of rice root and leaf to Mn and Se toxicity. This may also be related to differences in gene structure and motifs. In follow-up studies, these highly differentially expressed genes are worthy of further attention.

The ZIP family plays a crucial role in the transportation of indispensable micronutrients and heavy metal elements, such as Zn, Cu, Mn, and Fe [61]. ZIP transporters, containing CAX, NRAMP, CDF, HM-ATPase, and CTR, also impact ion homeostasis in plants by interacting with those proteins in overexpressed and mutated plants [14,62]. Studies have shown that plant ZIP participates in the differential regulation of all kinds of metal ions [63]. ZIP proteins have eight transmembrane domains with potential heavy metal binding domains, facilitating the transport of heavy metal ions into the cytoplasm [63].

In *A. thaliana*, AtIRT1 was the first confirmed ZIP protein [64]. It enables Arabidopsis to absorb Zn, Ni, and Cd from the rhizosphere to root cells [64]. Overexpression of *AtIRT1* in *A. thaliana* increases sensitivity to Cd, whereas the mutant *irt1-1* shows reduced sensitivity to Cd [65]. Additionally, Zn, Fe, and Mn deficiencies lead to decreased transcript abundance of *AtZIP2* in roots and buds [66]. BcIRT1 and BcZIP2 have been found to promote the accumulation of heavy metal ions, potentially transporting Cd^2+^, Mn^2+^, Zn^2+^, and Fe^2+^ [67]. These studies collectively highlight the essential role of ZIP family genes in root metal uptake, as well as in metal transport and distribution.

Mn is an indispensable chemical element in nearly all creatures and is involved in the regulation of many life activities [1]. In *A. thaliana*, it is estimated that 398 enzymes contain Mn at metal binding sites, with 20% demonstrating experimental evidence for the requirement of Mn as a cofactor. Notably, Mn can be substituted by other elements, such as Ca, Co, Cu, Mg, or Zn, in many enzymes [6].

Mn poisoning arises once the soluble Mn level in soil surpasses the proper concentration needed by plants [68]. Excessive Mn negatively impacts plant photosynthetic efficiency, resulting in the formation of Mn oxide spots, shrinkage of old leaf veins, leaf conduction tissue necrosis, and the leaves turning yellow. These detrimental effects cause a significant decline in plant biomass, with aboveground and underground dry weights decreasing by as much as 67% and 62%, respectively [68]. Ultimately, Mn toxicity severely hinders crop growth, development, and yield [69].

Low concentrations of Se have been found to protect plants from various abiotic stresses and enhance their tolerance to heavy metals [70]. This is attributed to the fact that Se improves plant antioxidant capacity, reduces reactive oxygen species (ROS), and limits lipid peroxidation [70]. However, the specific physiological mechanism by which excess Se affects plants has yet to be fully understood. At high doses, Se acts as a pro-oxidant and can cause damage to plants [71]. At present, the mechanism of Se–Mn interaction is still unclear. There are few studies on whether Se and Mn have some consistent regulatory mechanism, and whether Se can directly or indirectly affect Mn regulation-related genes. The results of this study indicate that OsZIPs may not only have a response to manganese toxicity stress, but also have a response to selenium poisoning. And this response may be more intense than manganese toxicity stress, which may be an unknown stress response mechanism, but the specific molecular regulatory mechanism is still unclear and needs further research. This study provides some insights for understanding the survival mechanism of rice under stress conditions such as excessive Mn or Se and the mechanism of the Se–Mn interaction, and can provide a theoretical basis for rice molecular breeding to improve stress resistance and yield. This is conducive to improving the economic value of rice, but also to ensuring food security and sustainable agricultural development.

## 5. Conclusions

This study identified 16 *OsZIPs* in the rice genome using multiple analytical methods. The physicochemical characters, structure of genes, phylogeny tree, and genetic expression patterns of the *OsZIPs* were confirmed. The phylogenetic evolution of these *OsZIPs* was categorized into three groups. The distribution of *OsZIPs* in all three taxa implies their similarity to the ZIPs of other species. Analysis of the conserved motifs in the structure of the OsZIPs indicated potential functional variations among them. Cis-element analysis of *OsZIPs* revealed their involvement in regulating plant hormone response factors. The qRT–PCR results demonstrated that the expression levels of *OsZIPs* in rice root and leaf significantly differed under respective Mn and Se toxicity stress, indicating their active response to these stresses. These findings provide a foundation for further understanding the role of *OsZIPs* in signal transduction during the response of rice to Mn or Se toxicity stress.

## Figures and Tables

**Table 1:** Summary of basic information about *OsZIPs*.

Gene Number	Gene ID	Chr	CDS (bp)	Portion Length	MW (kda)	PI	Subcellular Prediction
*OsZIP1*	LOC_Os01g74110.1	01	1059	352	37.45	8.89	Cell membrane [48]
*OsZIP2*	LOC_Os03g29850.1	03	1077	358	36.65	6.07	Cell membrane [48]
*OsZIP3*	LOC_Os04g52310.1	04	1095	364	38.14	8.75	Cell membrane [48]
*OsZIP4*	LOC_Os08g10630.1	08	1191	396	39.97	8.29	Cell membrane [48]
*OsZIP5*	LOC_Os05g39560.1	05	1062	354	36.76	6.85	Cell membrane [48]
*OsZIP6*	LOC_Os05g07210.1	05	1188	395	41.33	6.82	Cell membrane [48]
*OsZIP7*	LOC_Os05g10940.1	05	1155	384	39.73	7.06	Cell membrane [48]
*OsZIP8*	LOC_Os02g10230.1	02	831	276	29.28	7.88	Cell membrane [48]
*OsZIP9*	LOC_Os05g39540.1	05	1089	362	37.90	6.41	Cell membrane [48]
*OsZIP10*	LOC_Os06g37010.1	06	1215	404	41.53	7.03	Chloroplast [48]
*OsZIP11*	LOC_Os05g25194.1	05	1734	577	60.36	8.01	Cell membrane [NCBI]
*OsZIP12*	LOC_Os03g46470.1	03	1125	374	39.06	8.69	Cell membrane [NCBI]
*OsZIP13*	LOC_Os07g12890.1	07	1173	390	40.26	6.79	Cell membrane [NCBI]
*OsZIP14*	LOC_Os08g36420.1	08	1497	498	53.58	6.70	Cell membrane [NCBI]
*OsZIP15*	LOC_Os03g46454.1	03	705	234	24.15	8.04	Cell membrane [NCBI]
*OsZIP16*	LOC_Os08g01030.1	08	849	282	30.14	6.51	Cell membrane [NCBI]

## Data Availability

Data is contained within the article or Supplementary Material. The original contributions presented in the study are included in the article/Supplementary Material, further inquiries can be directed to the corresponding author/s.

## Supplementary Materials

The following supporting information can be downloaded at: https://www.mdpi.com/article/10.3390/genes15060696/s1, Table S1: the sequences of the amino acid in OsZIPs were compared with those of soybean (*G. max*), Arabidopsis thaliana, and maize (*Z. mays*); Table S2: the NSP1040 nutrient solution composition; Table S3: the qRT–PCR primers.
