# Supplementary material for: Genome-Wide Identification of OsZIPs in Rice and Gene Expression Analysis under Manganese and Selenium Stress
Source: Genes (Basel). 2024 May 27;15(6):696. doi: 10.3390/genes15060696 (PMC11202597; doi:10.3390/genes15060696)
Supplement: Supplementary file 1 [file genes-15-00696-s001.zip › Table S1.pdf]

Table S1 Species and ids of phylogenetic tree

Gene number	Gene ID	Portin Length
OsZIP1	LOC_Os01g74110.1	352
OsZIP2	LOC_Os03g29850.1	358
OsZIP3	LOC_Os04g52310.1	364
OsZIP4	LOC_Os08g10630.1	396
OsZIP5	LOC_Os05g39560.1	354
OsZIP6	LOC_Os05g07210.1	395
OsZIP7	LOC_Os05g10940.1	384
OsZIP8	LOC_Os02g10230.1	276
OsZIP9	LOC_Os05g39540.1	362
OsZIP10	LOC_Os06g37010.1	404
OsZIP11	LOC_Os05g25194.1	577
OsZIP12	LOC_Os03g46470.1	374
OsZIP13	LOC_Os07g12890.1	390
OsZIP14	LOC_Os08g36420.1	498
OsZIP15	LOC_Os03g46454.1	234
OsZIP16	LOC_Os08g01030.1	282
AtZIP1	AT3G12750.1	355
AtZIP2	AT5G59520.1	353
AtZIP3	AT2G32270.1	339
AtZIP4	AT1G10970.1	408
AtZIP5	AT1G05300.1	360
AtZIP6	AT2G30080.1	341
AtZIP7	AT2G04032.1	365

AtZIP8	AT5G45105.2	299
AtZIP9	AT4G33020.1	344
AtZIP10	AT1G31260.1	364
AtZIP11	AT1G55910.1	326
AtZIP12	AT5G62160.1	355
AtZIP13	AT4G19690.2	347
AtZIP14	AT4G19680.2	350
AtZIP15	AT1G60960.1	425
GmZIP1	Glyma.20g063100	354
GmZIP2	Glyma.08g164400	361
GmZIP3	Glyma.15g262800	359
GmZIP4	Glyma.13g004400	347
GmZIP5	Glyma.17g228600	393
GmZIP6	Glyma.11g169300	326
GmZIP7	Glyma.14g196200	324
GmZIP8	Glyma.04g051100	289
GmZIP9	Glyma.18g060300	328
GmZIP10	Glyma.14g094900	281
GmZIP11	Glyma.06g052000	478
GmZIP12	Glyma.08g328000	349
GmZIP13	Glyma.18g078600	360
GmZIP14	Glyma.15g036200	345
GmZIP15	Glyma.15g036300	342
GmZIP16	Glyma.13g338300	350
GmZIP17	Glyma.13g340900	276
GmZIP18	Glyma.15g033500	276

GmZIP19	Glyma.11g132500	272
GmZIP20	Glyma.12g056900	240
GmZIP21	Glyma.16g221000	288
GmZIP22	Glyma.09g271900	598
GmZIP23	Glyma.18g217100	598
ZmZIP1	NM_001137726	490
ZmZIP2	NM_001159169	359
ZmZIP3	NM_001155536	367
ZmZIP4	HM048832	386
ZmZIP5	NM_001154257	402
ZmZIP6	NM_001156151	396
ZmZIP7	NM_001157018	387
ZmZIP8	NM_001154769	396
ZmZIP9	NM_001158638	381
