# Supplementary material for: Genome-Wide Identification of OsZIPs in Rice and Gene Expression Analysis under Manganese and Selenium Stress
Source: Genes (Basel). 2024 May 27;15(6):696. doi: 10.3390/genes15060696 (PMC11202597; doi:10.3390/genes15060696)
Supplement: Supplementary file 1 [file genes-15-00696-s001.zip › Table S2.pdf]

Table S2 Manganese deficiency Yoshida nutrient solution

Ingredient	Concentration(mg/L)
NH_4NO_3	114.36
NaH_2PO_4	38.75
K_2SO_4	89.22
$CaCl_2$	110.76
$MgSO_4$	197.76
$(NH_4)_6 \cdot MO_7O_{24} \cdot 4H_2O$	0.093
H_3BO_3	1.168
$ZnSO_4 \cdot 7H_2O$	0.044
$CuSO_4 \cdot 5H_2O$	0.039
$FeCl_3$	5.775
$C_6H_8O_7 \cdot H_2O$	14.875
