# Supplementary material for: Genome-Wide Identification of OsZIPs in Rice and Gene Expression Analysis under Manganese and Selenium Stress
Source: Genes (Basel). 2024 May 27;15(6):696. doi: 10.3390/genes15060696 (PMC11202597; doi:10.3390/genes15060696)
Supplement: Supplementary file 1 [file genes-15-00696-s001.zip › Table S3.pdf]

Table S3 Primers of *OsZIPs* family for qRT-PCR detection

Primer name	Gene ID	Forward Sequence (5' to 3')	Reverse Sequence (5' to 3')
OsActin	LOC_Os03g50885	TTCCAGCCTT CCTTCATA	AACGATGTTGCCATATAGAT
OsZIP1	LOC_Os01g74110.1	TCTGTTTCCACTCCGTCTTT	GAATATCTTGTGCAGCCCGA
OsZIP2	LOC_Os03g29850.1	CACCGGCATCTTCATCTACG	AGGTGTCCCAGATCATGACG
OsZIP3	LOC_Os04g52310.1	CCAGTTCTTTGAAGGTGTAGG	CGGGTTATTGAAATCTGTTGCT
OsZIP4	LOC_Os08g10630.1	GCGACTTTCTTCTCCCTCAC	GGCAAGCAGAGACATCATCC
OsZIP5	LOC_Os05g39560.1	CAGGCAAAGTTCCGTGTCAG	CGCCATGTAGACGAGTATCC
OsZIP6	LOC_Os05g07210.1	GAGGAGGAAGCAGAAGATGG	GTCACCGAGAACATTACGCA
OsZIP7	LOC_Os05g10940.1	GGACGACAACGACAACAAGG	CATCTCAAGTATCTGCGACACC
OsZIP8	LOC_Os02g10230.1	CCTCAGCACGTCCATCGG	GGCCCTTCATGGATCCCAAA
OsZIP9	LOC_Os05g39540.1	CGCCTACAATGAGAATAGCC	TCAAGCCCAAATACCAAGCA
OsZIP10	LOC_Os06g37010.1	GGGAATTGTATCTCACTCGGTC	TAGCAAAGAAGAAAGCCATCAG
OsZIP11	LOC_Os05g25194.1	CACAACTCAGAAGATACAGCAG	GGAGGAATAAGGTGGTAAATAGAC
OsZIP12	LOC_Os03g46470.1	GAACCGCGTCGTCGTTCAG	CCATCCCCTCGAACATCTGG
OsZIP13	LOC_Os07g12890.1	CGGCATCGTGTCATCTCTCA	CCTCACCTTGAACTTCGCCT
OsZIP14	LOC_Os08g36420.1	CACCACCATCACCATGAACAC	CCTGGAAGAAGATTACTGGGAG
OsZIP15	LOC_Os03g46454.1	CGTTCCACACGCGGGGCAGCAAG	TGCGCCTTGGTGGTGTTACCG
OsZIP16	LOC_Os08g01030.1	ATTGGTGGCCTTAGCACCTC	AAGAAATGCCGCAGTACCCA
